# White matter integrity and motor function: a link between cerebral myelination and longitudinal changes in gait speed in aging

**DOI:** 10.1007/s11357-024-01392-w

**Published:** 2024-10-30

**Authors:** Zhaoyuan Gong, Mary E. Faulkner, Mohammad A. B. S. Akhonda, Alex Guo, Jonghyun Bae, John P. Laporte, Sarah Church, Jarod D’Agostino, Jan Bergeron, Christopher M. Bergeron, Luigi Ferrucci, Mustapha Bouhrara

**Affiliations:** 1 Laboratory of Clinical Investigation, National Institute on Aging, National Institutes of Health, BRC 05C-222, 251 Bayview Blvd., Baltimore, MD 21224 USA; 2 Clinical Research Core, National Institute on Aging, National Institutes of Health, Baltimore, MD 21224 USA; 3 Translational Gerontology Branch, National Institute on Aging, National Institutes of Health, Baltimore, MD 21224 USA

**Keywords:** Gait speed, Myelin, White matter, Magnetic resonance imaging, Motor function, Longitudinal data

## Abstract

Gait speed is a robust health biomarker in older adults, correlating with the risk of physical and cognitive impairments, including dementia. Myelination plays a crucial role in neurotransmission and consequently affects various functions, yet the connection between myelination and motor functions such as gait speed is not well understood. Understanding this link could offer insights into diagnosing and treating neurodegenerative diseases that impair mobility. This study analyzed 437 longitudinal observations from 138 cognitively unimpaired adults, aged 22 to 94 years, to investigate the relationship between myelin content and changes in gait speed over an average of 6.42 years. Myelin content was quantified using a novel multicomponent magnetic resonance relaxometry method, and both usual and rapid gait speeds (UGS, RGS) were measured following standard protocols. Adjusting for covariates, we found a significant fixed effect of myelin content on UGS and RGS. Longitudinally, lower myelin content was linked to a greater decline in UGS, particularly in brain regions associated with motor planning. These results suggest that changes in UGS may serve as a reliable marker of neurodegeneration, particularly in cognitively unimpaired adults. Interestingly, the relationship between myelin content and changes in RGS was only observed in a limited number of brain regions, although the reason for such local susceptibility remains unknown. These findings enhance our understanding of the critical role of myelination in gait performance in unimpaired adults and provide evidence of the interconnection between myelin content and motor function impairment.

## Introduction

Gait speed is a straightforward yet powerful measure of motor performance. It is increasingly recognized not just as a measure of mobility but as an indicator of overall health, predicting outcomes such as functional decline, the risk of falls, hospitalization, disability, and even mortality [1–7]. Gait speed reflects the integrity of multiple physiological systems, including the musculoskeletal, cardiovascular, and nervous systems. In the context of aging, gait speed is also closely related to cognitive function. Slower gait speeds have been associated with an increased risk of cognitive decline and dementia, particularly in older adults [8–13]. As such, it has become a valuable tool in clinical settings for identifying older adults at risk of adverse health outcomes and guiding interventions to maintain mobility and cognition.

Recent research, primarily based on cross-sectional studies, has revealed a relationship between abnormal gait speed and age-related changes in both the macro- and microstructures of gray and white matter, particularly in brain regions critical for mobility [14, 15]. However, the extent to which deterioration in brain white matter, especially myelin, contributes to poor gait performance over time remains unclear. A deeper understanding of this relationship could be achieved through longitudinal analyses, which would provide valuable insights into the temporal dynamics of white matter loss and gait speed decline across the lifespan. Such knowledge could help optimize targeted interventions and preventive strategies against age-related motor function decline and neurodegenerative diseases such as Alzheimer’s disease.

Previous studies utilizing structural magnetic resonance imaging (MRI) and diffusion tensor imaging (DTI) have comprehensively examined the relationships between neurodegeneration, specifically the loss of white matter microstructural integrity, and gait speed decline. For example, Callisaya et al. found significant longitudinal associations between changes in brain structures, including white matter atrophy, hippocampal atrophy, and white matter lesion progression, and the decline in gait speed [16]. Similarly, Lee et al. reported a significant longitudinal association between changes in overall gray and white matter volumes and gait speed [17]. Various studies have also explored the relationship between gait speed decline and neuropathology progression. For instance, Sullivan et al. found that gait speed decline was associated with white matter hyperintensities, beta-amyloid burden, and markers of cerebrovascular disease and Alzheimer’s disease [18]. Furthermore, van der Holst et al. demonstrated that declines in gait speed in older adults with cerebral small vessel disease were associated with white matter atrophy and loss of integrity, as indicated by increased mean and radial diffusivities and decreased fractional anisotropy [19].

Given the well-known limitations of DTI, several studies have implemented more advanced imaging techniques. For instance, using diffusion kurtosis imaging (DKI) in relapsing–remitting multiple sclerosis patients, Nygaard et al. found that mean diffusivity in cortical gray matter was significantly associated with complex walking functions and walking capacity [20]. Spampinato et al. reported that DKI metrics, particularly kurtosis metrics, are more sensitive than DTI metrics in detecting early corticospinal tract changes and predicting motor function recovery after acute ischemic stroke [21]. Additionally, other advanced diffusion biophysical models, such as neurite orientation dispersion and density imaging (NODDI), have shown significant correlations between gait parameters (e.g., stride length and stride time) and NODDI metrics, suggesting that white matter microstructural changes may contribute to gait impairment in Parkinson’s disease [22]. Moreover, in mild cognitive impairment (MCI) patients, Annweiler et al. used proton magnetic resonance spectroscopy and found that lower N-acetyl aspartate/creatine ratios were associated with higher stride time variability during dual-tasking, while higher choline/creatine ratios were linked to slower gait velocity [23]. Collectively, these studies establish gait speed as a reliable correlate of neurodegenerative processes associated with aging and disease. However, while sensitive to white matter degeneration, these diffusion techniques cannot directly measure myelin content due to technical and physiological constraints [24–29]. Therefore, despite the value of these investigations, the role of myelination, as measured with more specific techniques, on changes in motor dysfunction has not been clearly established.

Even with substantial research, a critical knowledge gap persists regarding the impact of myelin deterioration on changes in gait speed, particularly in normative aging. Previous studies assessed white matter integrity through conventional structural MRI and DTI methodology, which is hampered by inherent methodological limitations and biological confounders, including axonal degeneration, hydration, temperature, flow, macromolecular content, iron accumulation, and architectural features such as fiber fanning and crossing. On the other hand, advanced diffusion and spectroscopy studies remain scarce and are primarily focused on disease populations. Recently, advanced relaxometry methods have been introduced, offering unique, noninvasive approaches for in vivo measurement of myelin content, one of the major determinants of white matter integrity [30]. Specifically, the Bayesian Monte Carlo multicomponent–driven equilibrium single-pulse observation of T_1_ and T_2_ (BMC-mcDESPOT) analysis enables the quantification of local myelin water fraction (MWF), a direct measure of myelin content [31–34]. This technique has been widely used to demonstrate myelin loss in MCI and dementias [35], and has been instrumental in examining the associations between myelin content and various genetic, metabolic, and vascular risk factors [36–41], providing valuable insights into aging and neurodegeneration mechanisms. Recently, BMC-mcDESPOT revealed that lower cerebral myelination, as measured by MWF, is associated with lower gait speeds in cognitively unimpaired adults [42], further supporting the central role of myelin integrity in preserved motor function. However, since this study was cross-sectional, a potential causal link between cerebral myelin content and age-related gait speed decline is still to be established.

In this study, we used linear mixed-effects analyses to investigate the relationship between myelin content, measured via BMC-mcDESPOT, and longitudinal changes in gait speed measured over several years (mean ± SD = 6.42 ± 3.74 years). In particular, we conducted voxel-wise and regional analyses in cerebral lobar white matter using data from a cohort of 138 cognitively unimpaired adults spanning a broad age range from 22 to 94 years, after excluding datasets due to cognitive impairments or imaging artifacts. Our hypothesis posits that lower myelin content is associated with a steeper decline in gait speed. This investigation aims to contribute to a deeper understanding of the complex interplay between myelin content and gait speed, providing valuable insights into the possible causes of mobility changes during aging.

## Methods

### Subjects

The study uses data from participants in two ongoing aging-related research projects: the Baltimore Longitudinal Study of Aging (BLSA) and the Genetic and Epigenetic Signatures of Translational Aging Laboratory Testing (GESTALT), both conducted by the National Institute on Aging (NIA) in Baltimore, MD. Both studies share similar inclusion and exclusion criteria, focusing on the assessment of various physiological measures of aging. Participants underwent testing at the NIA’s clinical research unit and were excluded if they had metallic implants, or neurological or medical disorders. At the time of enrollment in our MRI protocol, participants were free of central nervous system disease (dementia, stroke, bipolar illness, epilepsy), severe cardiac disease, severe pulmonary disease, and metastatic cancer. Participants were enrolled in this study if they were free of significant cognitive impairment, namely they made less than three errors at the Blessed Information-Memory-Concentration (BIMC) Test and had a Clinical Dementia Rating (CDR) of zero. All experimental procedures adhered to ethical guidelines, receiving approval from the local Institutional Review Boards. Participants provided written informed consent at each visit, in line with the Declaration of Helsinki.

### Longitudinal gait speed measurement

Gait speed was assessed through two measurements, i.e., usual gait speed (UGS) and rapid gait speed (RGS), both recorded in meters per second (m/s). UGS was measured by having participants walk at their self-reported usual, comfortable pace over a 6-m stretch in an uncarpeted corridor, starting from behind a taped line and timed from the first foot fall past this line until crossing the finish line. RGS was measured using a similar method, with participants instructed to walk as fast as possible without running. Both assessments involved two trials, and the fastest recorded value was used in subsequent analyses. These measurements were conducted in the same facility to ensure consistency and accuracy. We note that the gait speed was measured at and retrospectively to the single point MRI. Our goal is to investigate the association between differences in MWF and retrospective changes in gait speed.

### Cross-sectional MRI acquisition

All MRI scans were performed on a 3-T whole body Philips MRI system (Achieva, Best, The Netherlands) using the internal quadrature body coil for transmission and an eight-channel phased-array head coil for signal acquisition. Following the BMC-mcDESPOT imaging protocol [43], three sequences were acquired to calculate MWF: 3D SPGR images were acquired with flip angles (FAs) of [2 4 6 8 10 12 14 16 18 20]°, echo time (TE) of 1.37 ms, and repetition time (TR) of 5 ms. 3D bSSFP images were acquired with FAs of [2 4 7 11 16 24 32 40 50 60]°, TE of 2.8 ms, and TR of 5.8 ms. The bSSFP images were acquired twice with radiofrequency excitation pulse phase increment of 0 and π to account for off-resonance effects. All SPGR and bSSFP images were acquired with an acquisition matrix of 150 × 130 × 94, and a voxel size of 1.6 mm × 1.6 mm × 1.6 mm. To correct for excitation radiofrequency inhomogeneity, B_1_, we used the double-angle method (DAM) by acquiring two fast spin-echo images with FAs of 45° and 90°, TE of 102 ms, TR of 3000 ms, and acquisition voxel size of 2.6 mm × 2.6 mm × 4 mm [44]. All images were acquired with a field of view of 240 mm × 208 mm × 150 mm, and reconstructed to voxel size of 1 mm × 1 mm × 1 mm.

### Image processing

For each participant, a whole-brain MWF map was generated from the SPGR, bSSFP, and B_1_ images using BMC-mcDESPOT [32–34]. Further, using the FSL software [45], the averaged SPGR image over FAs underwent nonlinear registration to the Montreal Neurological Institute (MNI) standard space, with the computed transformation matrix then applied to the corresponding MWF maps. In addition to a voxel-wise statistical analysis using registered MWF maps, we also conducted secondary regional statistical analysis. Our regions of interest (ROIs) included cerebellar WM and the WM of the four cerebral lobes, namely the frontal, parietal, temporal, and occipital lobes. We also investigated whole brain (WB) WM. These six WM ROIs were defined using the MNI structural atlas in FSL [45]. Within each ROI, the mean MWF values were calculated.

### Statistical analysis

Linear mixed-effects models were used to investigate associations between regional or voxel-wise MWF values and longitudinal changes in gait speed. The dependent variable was each of the corresponding *z*-scored longitudinal gait speed (UGS or RGS). Age at MRI, Time-to-MRI, sex, race, years of education (EDY), regional or voxel-wise MWF value, and MWF × time interaction were included as independent variables. A random intercept was included per participant. The explicit regression model was:

$$\text{GS}{ }_{ij}={\upbeta }_{0}+{\upbeta }_{\text{age}}\times {\text{age}}_{i}+{\upbeta }_{\text{sex}}\times {\text{sex}}_{i}+{\upbeta }_{\text{race}}\times {\text{race}}_{i}+{\upbeta }_{\text{EDY}}\times {\text{EDY}}_{i}+{\upbeta }_{\text{time}}\times {\text{time}}_{ij}+{\upbeta }_{\text{MWF}}\times {\text{MWF}}_{i}+{\upbeta }_{\text{time }\times \text{ MWF}}\times {\text{MWF}}_{i}\times {\text{time}}_{ij}+{\upepsilon }_{ij}+{b}_{i},$$

where GS_*ij*_ is the gait speed (UGS or RGS) *z* score of subject *i* at timepoint *j*, age_*i*_ is the age of subject *i* (in years) at MRI, MWF_*i*_ is the MWF value of subject *i*, time_*ij*_ is the time to MRI of subject *i* at time-point *j*, *b*_*i*_* ∼ N*(0*, σ*_*b*_^*2*^) is the random intercept for subject *i*, and *ϵ*_*ij*_* ∼ N*(0, *σ*_*ϵ*_^*2*^) is the residual. We note that the main parameters of interest in this analysis are β_MWF_, reflecting the adjusted fixed effect of MWF on gait speed; β_time_, reflecting the expectation of annual longitudinal change in gait speed; and β_time×MWF_, reflecting the expectation of the difference in the longitudinal change in gait speed per unit difference in MWF. Statistical significance for regional analysis was determined using Benjamini–Hochberg (BH) method corrected *P* < 0.05, while for voxel-wise analysis, significance was defined as uncorrected *P* < 0.01 with a cluster size exceeding 400 voxels.

## Results

**Fig. 1:**
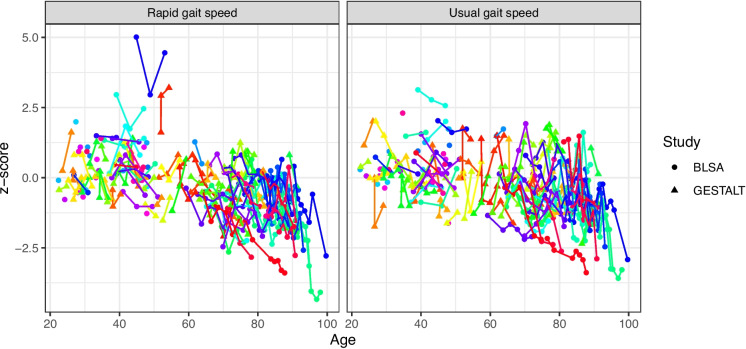
Visualization of the longitudinal gait speed measures in z-score for each participant. Both rapid gait speed and usual gait speed are shown. Each subject is color coded, line connected and denoted with either a circle or triangle for BLSA or GESTALT study, respectively

Table 1 provides the demographic characteristics of the final study cohort. After excluding visits with missing gait speed data, severe motion artifacts in MR images, or cognitive impairments, the final cohort consisted of 138 cognitively unimpaired volunteers, aged 22 to 94 years. Of these participants, 61 (44.2%) were female, and 27 (19.6%) were Black. No significant age difference was observed between males and females. Gait speed measurements were collected over an average follow-up period of 6.42 ± 3.74 years, resulting in a total of 437 measures. The average UGS was 1.25 ± 0.22 m/s, and the average RGS was 1.88 ± 0.32 m/s (Table 1). Additionally, the number of subjects with different numbers of gait speed measurements is explicitly listed in Table 1. Specifically, the breakdown of longitudinal gait speed measures for each subject is shown, illustrating how many participants had one, two, three, and more than three measurements. This breakdown, along with Fig. 1, provides a visual representation of the overall decrease in UGS and RGS with age across the study cohort, as expected.

**Table 1:** Participant demographics

Characteristic	All participant (*n* = 138)				BLSA (*n* = 85)				GESTALT (*n* = 53)			
Age at MRI scan (years), mean (SD)	58.94 (21.2)				60.2 (22.6)				56.9 (18.8)			
Sex Female, *n* (%)	61 (44.2%)				39 (45.9%)				22 (41.5%)			
Education, mean (SD) (years)	16.30 (2.79)				16.73 (2.74)				15.62 (2.74)			
Number of gait assessments,	1	2	3	> 3	1	2	3	> 3	1	2	3	> 3
Counts	29	31	42	36	29	13	9	34	0	18	33	2
Duration of gait speed assessments (years), median (mean, SD)	6.16 (6.42, 3.74)				8.18 (7.75, 3.80)				4.09 (3.69, 1.41)			
UGS score, mean (SD) in m/s	1.25 (0.22)				1.26 (0.21)				1.23 (0.22)			
RGS score, mean (SD) in m/s	1.88 (0.32)				1.89 (0.36)				1.86 (0.25)			
Race, *n* (%)												
Race, Black	27 (19.6%)				20 (23.5%)				7 (13.2%)			
Race, White	95 (68.8%)				53 (62.4%)				42 (79.2%)			
Race, Other	16 (11.6%)				12 (14.1%)				4 (7.6%)			

*MRI* magnetic resonance imaging, *SD* standard deviation, *BLSA* Baltimore Longitudinal Study of Aging, *GESTALT* Genetic and Epigenetic Signatures of Translational Aging Laboratory Testing, *UGS* usual gait speed, *RGS* rapid gait speed

**Fig. 2:**
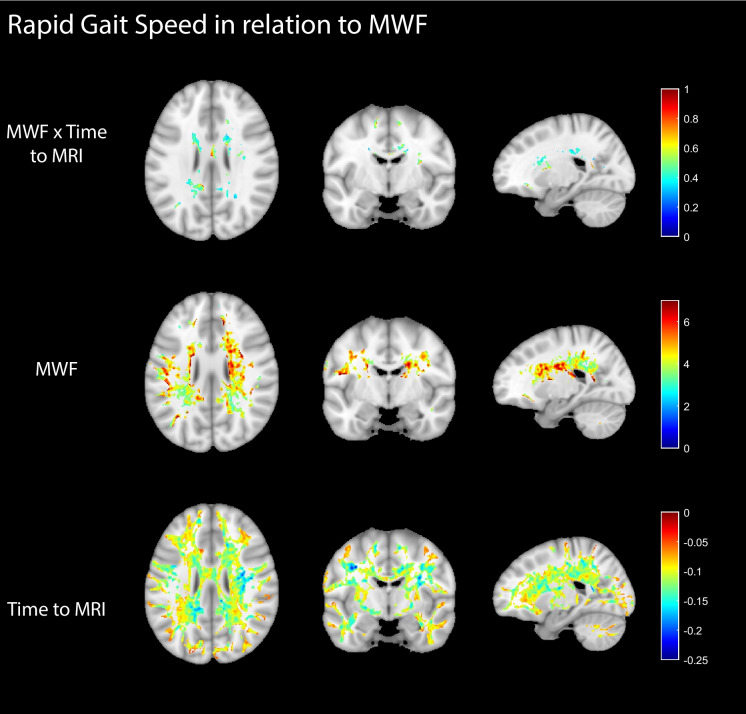
Regression coefficient maps illustrating the effects of MWF × Time-to-MRI, MWF, or Time-to-MRI terms in the linear mixed-effects models for RGS. Only statistically significant voxels are displayed (uncorrected *P* < .01, cluster size > 400 voxels). The fixed effect MWF term and the longitudinal Time-to-MRI term exhibited the opposite but both significant correlations with RGS. Interestingly, the MWF × Time-to-MRI interaction terms showed minimal correlation with RGS, indicating the minimal effect of MWF on longitudinal changes in RGS

**Fig. 3:**
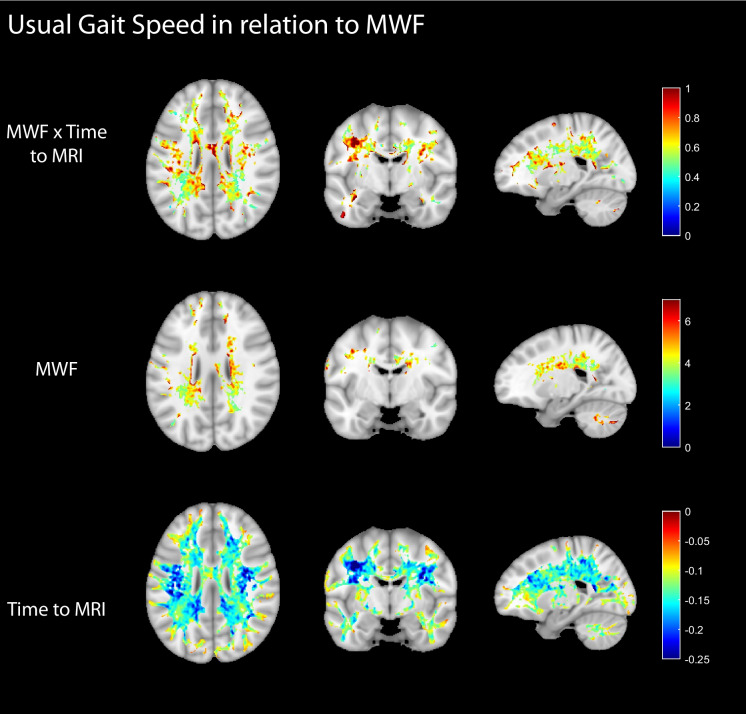
Regression coefficient maps illustrating the effects of MWF × Time-to-MRI, MWF, or Time-to-MRI terms in the linear mixed-effects models for UGS. Only statistically significant voxels are displayed (uncorrected *P* < .01, cluster size > 400 voxels). Several significant clusters were observed for the fixed effect MWF term, whereas the longitudinal Time-to-MRI term exhibited significant negative correlations with UGS across most brain regions. Importantly, the MWF × Time-to-MRI interaction terms revealed widespread regions of positively correlated significant clusters within cerebral white matter for UGS. This positive correlation demonstrated the slower longitudinal decline of UGS when the baseline MWF level is higher

Figures 2 and 3 display regression coefficient maps, derived from the voxel-wise analysis, illustrating the effects of the MWF × Time-to-MRI, MWF, Time-to-MRI terms incorporated in the linear mixed-effects models, for RGS (Fig. 2) or UGS (Fig. 3). The maps only include coefficients for voxel that are statistically significant at an uncorrected threshold of *P* < 0.01 and a minimum cluster size of 400 voxels. As expected, the longitudinal Time-to-MRI term exhibited strong and consistent negative correlations with UGS and RGS, indicating that longer periods spanned from the initial gait speed assessment to the baseline MRI scan were associated with further declines in gait speeds. This association was statistically significant in most white matter regions. Further, the fixed effect of MWF showed that participants with lower myelin content (lower MWF values) exhibited lower UGS and RGS. Importantly, the MWF × Time-to-MRI interaction terms revealed a pattern of numerous significantly positively correlated clusters within several white matter regions for UGS, suggesting that higher myelin content was associated with a less steep longitudinal decline in UGS over time. Interestingly, this association was limited to scattered clusters of voxels for RGS.

**Fig. 4:**
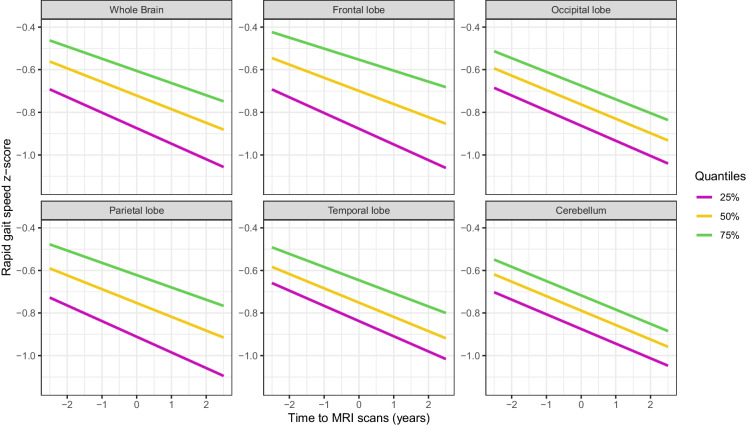
Panels depict corresponding representative longitudinal RGS decline curves obtained from the linear mixed-effects regression models. The magenta, yellow, and green lines represent the longitudinal changes in RGS at low, median, and high MWF values, respectively. It is evident that the rate of longitudinal RGS decline is not affected by the baseline MWF level

**Fig. 5:**
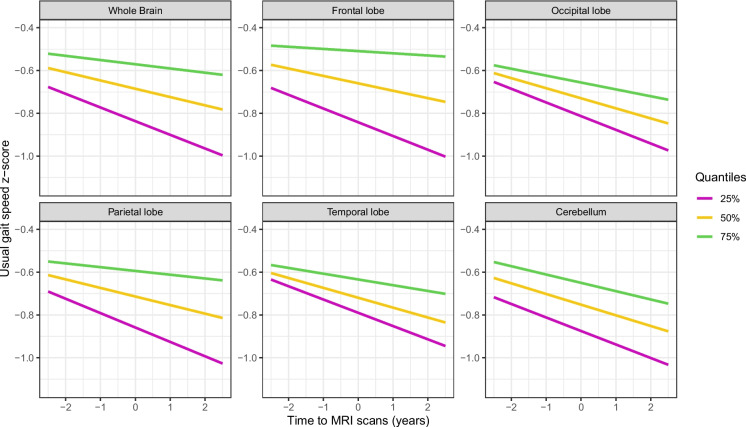
Panels depict corresponding representative longitudinal UGS decline curves obtained from the linear mixed-effects regression models. The magenta, yellow, and green lines represent the longitudinal changes in UGS at low, median, and high MWF values, respectively. It is evident from the curves that lower MWF values were associated with steeper declines in UGS. These associations are significant for the whole brain, frontal, occipital, parietal and temporal white matter

Six ROI-based regional analyses were conducted to assess the association between regional MWF and changes in gait speeds. Figures 4 and 5 show representative longitudinal decline curves of RGS and UGS, respectively, obtained from the linear mixed-effects regression models. The lines illustrate the longitudinal changes in gait speed at low, median, and high MWF values, represented by magenta, yellow, and green colors, respectively. Consistent with the voxel-wise analysis, the results of this analysis indicate that lower regional MWF values were associated with a steeper decline in UGS in all ROIs examined except for cerebellum. These associations were not observed for RGS. We also observed that males had a significantly higher rapid gait speed than females over the study period, even after adjusting for other covariates. Detailed statistical results are presented in Tables 2 and 3.

**Table 2:** ROI wise statistical results for rapid gait speed

		Whole brain	Frontal	Occipital	Parietal	Temporal	Cerebellum
*MWF*	β =	**6.862**	**6.974**	4.829	4.381	**6.337**	4.852
	*P* =	0.010	0.006	0.043	0.052	0.012	0.070
	*P*^*BH*^ =	**0.024**	**0.024**	0.062	0.062	**0.024**	0.070
Time-to-MRI	β =	** − 0.134**	** − 0.148**	** − 0.108**	** − 0.093**	** − 0.124**	** − **0.075
	*P* =	0.004	0.001	0.013	0.035	0.005	0.067
	*P*^*BH*^ =	**0.010**	**0.006**	**0.019**	**0.042**	**0.010**	0.067
*MWF* × Time	β =	0.403	0.475	0.244	0.150	0.345	0.058
	*P* =	0.148	0.067	0.343	0.567	0.193	0.854
	*P*^*BH*^ =	0.386	0.386	0.514	0.680	0.386	0.854
Age	β =	** − 0.020**	** − 0.019**	** − 0.022**	** − 0.022**	** − 0.020**	** − 0.023**
	*P* =	< 0.001	< 0.001	< 0.001	< 0.001	< 0.001	< 0.001
	*P*^*BH*^ =	** < 0.001**	** < 0.001**	** < 0.001**	** < 0.001**	** < 0.001**	** < 0.001**
Sex	β =	**0.399**	**0.400**	**0.383**	**0.381**	**0.417**	**0.368**
	*P* =	0.001	0.001	0.002	0.002	0.001	0.003
	*P*^*BH*^ =	**0.003**	**0.003**	**0.003**	**0.003**	**0.003**	**0.003**

The significant *P*-values (*P*^*BH*^ < 0.05) and corresponding beta coefficients are highlighted in bold

**Table 3:** ROI wise statistical results for usual gait speed

		Whole brain	Frontal	Occipital	Parietal	Temporal	Cerebellum
*MWF*	β =	**6.790**	**7.155**	3.923	3.667	**5.793**	**6.919**
	*P* =	0.015	0.007	0.116	0.119	0.028	0.013
	*P*^*BH*^ =	**0.030**	**0.030**	0.119	0.119	**0.042**	**0.030**
Time-to-MRI	β =	** − 0.234**	** − 0.246**	** − 0.196**	** − 0.173**	** − 0.227**	** − 0.145**
	*P* =	0.000	0.000	0.000	0.001	0.000	0.004
	*P*^*BH*^ =	** < 0.001**	** < 0.001**	** < 0.001**	**0.002**	** < 0.001**	**0.004**
*MWF* × Time	β =	**1.128**	**1.162**	**0.884**	**0.741**	**1.087**	0.745
	*P* =	0.001	0.000	0.005	0.021	0.001	0.054
	*P*^*BH*^ =	**0.002**	**0.001**	**0.007**	**0.025**	**0.002**	0.054
Age	β =	** − 0.015**	** − 0.014**	** − 0.018**	** − 0.018**	** − 0.016**	** − 0.017**
	*P* =	< 0.001	< 0.001	< 0.001	< 0.001	< 0.001	< 0.001
	*P*^*BH*^ =	** < 0.001**	** < 0.001**	** < 0.001**	** < 0.001**	** < 0.001**	** < 0.001**
Sex	β =	0.152	0.153	0.131	0.135	0.163	0.127
	*P* =	0.236	0.230	0.308	0.291	0.210	0.311
	*P*^*BH*^ =	0.311	0.311	0.311	0.311	0.311	0.311

The significant *P*-values (*P*^*BH*^ < 0.05) and corresponding beta coefficients are highlighted in bold

## Discussion

Gait speed is considered one of the most robust biomarkers of health and function in older persons and a strong predictor of many adverse health outcomes [7, 46–50]. Usual gait speed mirrors everyday mobility, while rapid gait speed tests physical capability under challenge and provides information on resilience while uncovering sub-clinical mobility problems. Established benchmarks indicate an elevated risk at UGS < 1 m/s, and improvements in UGS lowers mortality risk [51, 52], while longitudinal RGS assessment predicts disability [1]. Several MRI-based studies have investigated the correlation between cerebral structural integrity and gait speed, both macroscopically and microscopically [16–19, 53–55]. However, most prior studies were cross-sectional and utilized non-specific MR imaging metrics that do not provide reliable, quantitative metrics of myelination. Our study focuses on the correlation between baseline myelin content, measured by MWF MRI, and longitudinal gait speed measurements. As expected, we found that in most individuals gait speed declines gradually in the period prior to the MRI evaluation, likely reflecting the natural aging process where physical functions deteriorate [56, 57]. This decline might also signal progressive changes in participants’ health or mobility that are not directly associated with myelin, aligning with the consensus that aging correlates with a gradual decrease in gait speed due to factors like muscle weakness, balance impairments, or neurological function degradation [58, 59]. MWF across several white matter regions was significantly and independently correlated with RGS and UGS, suggesting that better myelin integrity correlates with faster gait performance. Contrary to the previous cross-sectional study where no significant association between UGS and MWF was found [42], we demonstrated the sensitivity of both RGS and UGS to subtle changes in myelin integrity. It is worth noting that the inherent limitations of cross-sectional designs, which only provide a snapshot of the relationship between variables at a single point in time, may not capture the nuanced relationship between MWF and gait speed. In our longitudinal study, the fixed effect of MWF represents how baseline MWF is associated with overall gait speed across all time points in the study, averaged over the study period. Additionally, including random intercept in the model controls for inter-individual variability, isolating the MWF fixed effect to focus on the average relationship between baseline MWF and gait speed within individuals across time points.

Interestingly, the outcomes for the interaction between time and MWF diverged from the fixed effect of MWF. Only limited regional significance was found for RGS regarding the MWF × Time interaction term, whereas a widespread positive correlation between this interaction term and UGS was evident. These results indicate that higher MWF is significantly associated with slower UGS declines over time yet does not significantly relate to RGS changes over the same period. The voxel-wise analysis results were similar to the findings in the ROI analysis. Major white matter regions exhibited a similar RGS decline rate, suggesting a stable positive correlation between myelin integrity and RGS throughout the observed period. In contrast, lower MWF was significantly associated with a steeper decline of UGS in the whole brain, frontal, occipital, parietal, and temporal white matter, indicating a dynamic relationship between myelin integrity and UGS that grows more pronounced with aging [42]. This dynamic may reflect the cumulative effects of aging on neural and motor functions, with UGS increasingly dependent on the integrity of neural pathways influenced by myelination. The differences highlight that UGS and RGS may capture different aspects of gait that are influenced by neural integrity, with UGS being more sensitive to changes in brain structure and function that occur with aging. For RGS, other physiological factors, including muscle weakness, may become more critical determinants of performance over time, masking any changing impact of myelin integrity [60–62]. However, myelin integrity remains a key determinant of UGS performance, with its influence becoming more evident with aging. Further investigations in larger cohorts and using longer periods of gait speed assessment are still required to confirm or refute our findings.

We observed significant sex differences in rapid gait speed, but not in usual gait speed. Previous research indicates that sex differences in physical performance are influenced by factors such as body composition, muscle strength, and neuromuscular activation [63–66]. For instance, men typically have greater muscle mass and strength, which may enhance their performance in tasks requiring rapid gait speed [64]. Rapid gait speed is a more demanding condition that relies on strength and power, potentially explaining why significant differences emerge in this context, whereas usual gait speed may involve less intensity, resulting in less pronounced differences. However, the study did not control for other potential confounding factors, such as lifestyle habits (e.g., physical activity levels, diet) and comorbid conditions, that could influence gait speed. Additionally, while muscle strength is an important factor, other determinants like social and psychological factors (e.g., motivation, confidence) that may affect rapid gait performance were not examined. Further research is needed to explore these elements and their interactions to fully understand the underlying mechanisms behind sex differences in gait speed.

This study comes with limitations. Although the BLSA and GESTALT studies are comparable, the BLSA study benefits from a longer span and more frequent follow-up visits. Despite the abundant gait speed measurements across both studies, the advanced MRI scans were limited to a participant subset, constraining our final cohort size to 138. Moreover, the representation of subjects aged 50 to 65 was notably lower than other age groups, potentially limiting the generalizability of our findings to the broader population. Our sample also displayed a slight imbalance in gender and racial representation, emphasizing the need for more diverse cohorts. Furthermore, despite our method of myelin mapping being state-of-the-art, future validation of imaging biomarkers, more specifically and sensitively tied to biological markers like myelin, remains essential. Future research should also aim to refine the specificity of novel MRI imaging biomarkers while maintaining their sensitivity.

In conclusion, our study reveals a complex relationship between myelin integrity and gait speed in normative aging. MWF strongly correlates with gait speed across most white matter regions, indicating a direct link between neural health and physical capability. MWF also shows a nuanced association with UGS changes over time, highlighting the dynamic impact of aging on the association between myelin integrity and UGS. These findings emphasize gait speed measurement as a biomarker for functional health and neurological integrity in older adults and suggest the potential for targeted interventions to maintain or improve mobility and independence. Despite limitations, the study provides valuable and novel insights into aging, offering a foundation for future research in imaging biomarkers and the complex interplay between physical function and brain health in older adults.

## Data Availability

Data from the BLSA and GESTALT can be obtained through formal requests on the study website. Analysis code can be obtained upon request to the corresponding authors.
